# Socio-demographic inequalities in the uptake of Papanicolaou tests in Peru: analysis of the 2015-2017 Demographic and Family Health Survey

**DOI:** 10.4178/epih.e2020043

**Published:** 2020-06-18

**Authors:** Antonio Barrenechea-Pulache, Emmanuel Avila-Jove, Akram Hernández-Vásquez, Fernando M. Runzer-Colmenares

**Affiliations:** 1 Facultad de Medicina, Universidad Científica del Sur. Lima, Peru; 2 Universidad San Ignacio de Loyola, Vicerrectorado de Investigación, Centro de Excelencia en Investigaciones Económicas y Sociales en Salud. Lima, Peru

**Keywords:** Population characteristics, Socioeconomic factors, Uterine cervical neoplasmas, Papanicolaou test, Health surveys, Peru

## Abstract

**OBJECTIVES:**

This study was conducted to analyze the inequalities in Papanicolaou test (also referred to as the Pap smear) uptake according to the socio-demographic characteristics of Peruvian women 30 years to 59 years of age using information from the 2015-2017 Demographic and Family Health Survey (ENDES, acronym in Spanish).

**METHODS:**

This is an analytical, cross-sectional study based on information acquired from the 2015-2017 ENDES surveys. Socio-demographic characteristics were reported using absolute frequencies and weighted proportions with 95% confidence intervals, considering results with a p-value <0.05 as statistically significant. Concentration curves (CCs) and concentration indices (IndCs) were created based on the interaction of the wealth index and uptake of Pap smears, taking into account the different characteristics of the population studied for the measure of inequalities.

**RESULTS:**

All the CCs were distributed below the line of equality. Similarly, all the IndCs were higher than zero, indicating inequality in the uptake of Pap smears, favoring those with a higher wealth index. The highest IndC values were obtained from women aged 50-59 (IndC, 0.293), those who lived in the jungle (IndC, 0.230), and those without health insurance (IndC, 0.173).

**CONCLUSIONS:**

We found socio-demographic inequalities in the uptake of Pap smears in Peru, favoring women with a higher wealth index. More funding is needed to promote cervical cancer screening programs and to create systems that ensure equal access to healthcare in Peru.

## INTRODUCTION

Cervical cancer, which is associated with persistent infection by the human papillomavirus (HPV), is currently the third leading cause of women cancer death in the world, causing an estimated 311,365 deaths in 2018 [1]. Like all other cancers attributable to infection, cervical cancer has a greater incidence in low-income nations due to limitations in access to prevention and screening methods, among other factors [2,3]. Peru is no stranger to this reality, with cervical cancer being the second most frequent malignant neoplasm among women and the first oncologic cause of death among women of 15 years to 44 years of age [4].

Among the various methods included in the national policy for cervical cancer screening in Peru, the Papanicolaou test (also known as the Pap test or Pap smear) is one of the most cost-effective and commonly used. Since the implementation of the *Plan Esperanza* in 2012, the government has carried out a series of changes that have allowed over 4 million women to receive a Pap test by the year 2015 [5]. However, the 2017 Demographic and Family Health Survey (ENDES, acronym in Spanish) showed that 37.2% of Peruvian women had not had a Pap test in the 3 years leading up to the survey [6]. Thus, there continue to be barriers limiting the uptake of screening, thereby increasing the risk of developing cervical cancer.

Previous studies have described individual and geographic socio-demographic characteristics that are related to the uptake of the Pap smear. Individual characteristics such as older age, poor education, and lower-income are related to low screening rates while having health insurance and a higher wealth index are the main factors related to higher screening rates [7,8]. In regard to geographic characteristics, living in a rural or remote area has shown to be a barrier to Pap test uptake [9,10]. Currently, the Peruvian national health survey limits itself to employing measures of central tendency to present descriptive information on Pap smear uptake [6,11], which does not fully represent the inequalities among the different actors of Peruvian society.

To date, there are scarce population-based studies that analyze the inequalities in the performance of Pap tests among Peruvian women. Thus, the objective of this article was to analyze the inequalities in Pap smear uptake according to the socio-demographic characteristics of Peruvian women 30 years to 59 years of age using information from the 2015-2017 ENDES surveys. By doing so, this research aims to help reduce the gaps in screening uptake, and thus, to avoid further deaths from a preventable disease.

## MATERIALS AND METHODS

### Design and study population

We performed a secondary analytical study including Peruvian women aged 30 years to 59 years who answered the ENDES questionnaires in the years 2015-2017. These questionnaires were based on clinical practice guidelines, which recommend that Pap smears be performed in sexually active women starting at age 30 with an interval of no more than 3 years [12,13]. The ENDES is an annual survey carried out by the *Instituto Nacional de Estadística e Informática* (INEI) of Peru with the objective of obtaining up-to-date information about the demographic dynamics and health condition of mothers, children younger than 5, and people older than 15 years of age who are residents of Peru. ENDES employs probabilistic, balanced, 2-stage, stratified, and cluster sampling to obtain an annual sample that is designed to yield representative estimates at national, departmental, and natural region levels. The ENDES database is free to access, and more details about the sampling process, characteristics of the study population, and estimation of the weighting factors used in the survey can be found in the technical report [14].

### Variables and measurements

Self-reporting of having undergone a Pap smear in the 3 years prior to the survey was considered as the dependent variable, while the wealth index of the participating women was considered as the independent variable of interest. This index is a measure of household wealth that is measured by assigning a score generated through the principal component analysis method, depending on the availability of goods, services, and housing characteristics [15]. In addition, to characterize the population, a wealth index categorized into 5 quintiles was used (the first quintile being that with the lowest level of well-being and quintile five indicating the highest level of well-being). The relationship of these variables is presented through a stratification according to the following characteristics of the population: (1) age group: women 30 to 39, 40 to 49, and 50 to 59 years old; (2) highest educational level reached: up to primary, secondary, and higher; (3) area of residence: rural or urban (the rural area of Peru is characterized by its low access of health services, due to the low supply of health facilities and high turnover of health professionals) [16]; (4) natural region of residence: coast, highlands and jungle (it should be noted that according to its natural regions, Peru is divided into the coast, where the largest population and the most developed cities are concentrated; the highlands, a region characterized by high levels of poverty and poor provision of health services corresponding to the Peruvian Andes; and the jungle, which is difficult to access due to natural geographic barriers typical of an Amazonian territory); (5) health insurance affiliation: *Seguro Integral de Salud* (SIS; for people in poverty and extreme poverty), Social Health Insurance (EsSalud; for dependent workers and their legal beneficiaries), others (police and armed forces healthcare and private sector, the latter of which consists of private clinics, private practice and other municipal or non-profit healthcare providers), and none [17]; (6) altitude above sea level of the housing conglomerate: 0-499 m above sea level (m. a. s. l.) (it should be emphasized that this altitude includes both the coast and much of the jungle), 500-1,499 m. a. s. l., 1,500-2,999 m. a. s. l., and 3,000 m or more a. s. l. The variables selected were based on previous studies and the Andersen conceptual framework for the study of access to healthcare [10,18-21].

### Statistical analysis

The databases included for each year of the survey were obtained from the INEI web (http://iinei.inei.gob.pe/microdatos/). They were combined for the analysis because the samples selected for the years 2015, 2016, and 2017 belonged to a single sampling frame.

The characteristics of the population are described as absolute frequencies and weighted proportions with 95% confidence intervals (CIs). The weighted proportions of participants who reported receiving a Pap test for each of the study variables with their respective 95% CIs are also described. Subsequently, the analysis of inequalities in the uptake of the Pap test was carried out based on concentration curves (CCs) and concentration indices (IndCs) following the World Bank guidelines [22]. In this study, CCs describe the relationship between the cumulative percentage of individuals who received a Pap test within the last 3 years and the cumulative percentage of the population distributed according to the wealth index, stratified according to selected characteristics of the population, in relation to the diagonal line of equality. Inequality is estimated according to the concavity of the curve; that is, the further the CC moves away from the line of equality, the greater the inequality in the relationship studied. A CC below the line of equality indicates a higher uptake percentage of the Pap test by the population that has higher wealth indices. In contrast, a CC above the equality line corresponds to a higher uptake percentage of the Pap test by the population with lower wealth indices. The IndC measures the magnitude of inequality. The values of the coefficient range from -1 to 1, with 0 being complete equality. A value is positive when the distribution of the variable studied is concentrated in the population with higher wealth indices and vice versa. For this purpose, the *Conindex* command was used [23].

Statistical processing and analyses were performed using Stata version 14.2 (StataCorp., College Station, TX, USA) specifying the survey sampling characteristics, which included the weighting factor and complex design of ENDES. A p-value < 0.05 was considered to indicate statistical significance for all statistical tests.

### Ethics statement

This study was approved by the Institutional Review Board (IRB) of the Universidad Científica del Sur (IRB approval No. 284-2018-PRE15). It is an analysis of a de-identified secondary dataset of the ENDES, which is freely and publicly available.

## RESULTS

**Figure 1.**
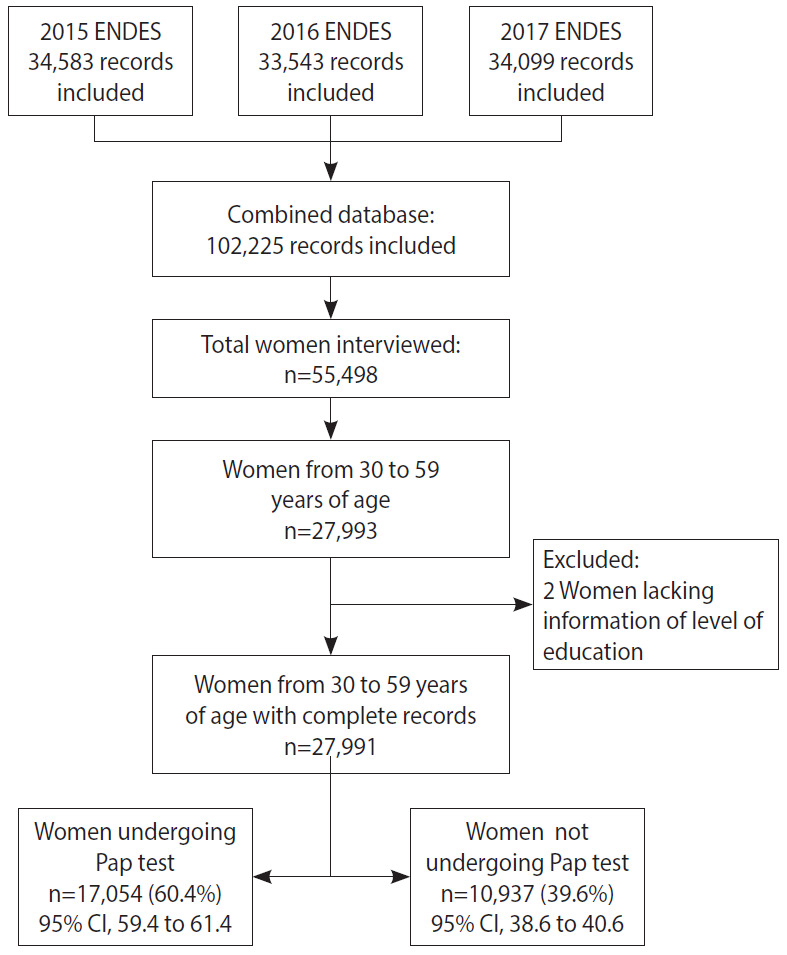
Flowchart of participants included in the study. during the 2015-2017 Demographic and Family Health Survey (ENDES, acronym in Spanish)

Data from a total of 27,991 women between 30 years and 59 years of age who participated in the ENDES 2015-2017 were included (Figure 1). Table 1 presents the characteristics of the women included in the study. The mean age of the women studied was 40.7± 8.3 years. The plurality was in the age range of 30-39 years (40.6%), had up to primary (35.8%) or secondary education (35.0%), and had health insurance through the SIS (47.7%). With regard to altitude, most lived within 499 m. a. s. l. (56.4%) and resided in the natural region of the coast (55.3%), while a minority lived in the jungle (14.0%). The respondents predominantly lived in urban areas (71.7%).

The characteristics of the women and percentages who received Pap smears in the last 3 years can be seen in Table 2. A Pap test was received by 60.4% of the respondents within the 3 years prior to the survey. The highest percentage of women who received a Pap test was within the age group of 30-39 years (66.3%), followed by the age group of 40-49 years (62.1%); 63.5% had a secondary education level or higher (69.9%), 74.4% had health insurance affiliated with EsSalud, and 71.5% belonged to the highest economic wealth quintile (V). Receiving Pap tests was more common among women who lived below 499 m. a. s. l. (63.7%), on the coast (65.5%), and in urban areas (63.7%). The women with the lowest uptake of the Pap test were those from 50-59 years of age (49.0%), with up to primary education (49.5%), and who belonged to the lowest economic wealth quintile (48.4%), lived at an altitude greater than 3,000 m. a. s. l. (52.3%), lived in the jungles (51.1%) and in rural areas (52.1%), and had no affiliation with health insurance (54.3%). All the characteristics evaluated presented statistically significant differences (p< 0.001).

**Figure 2.**
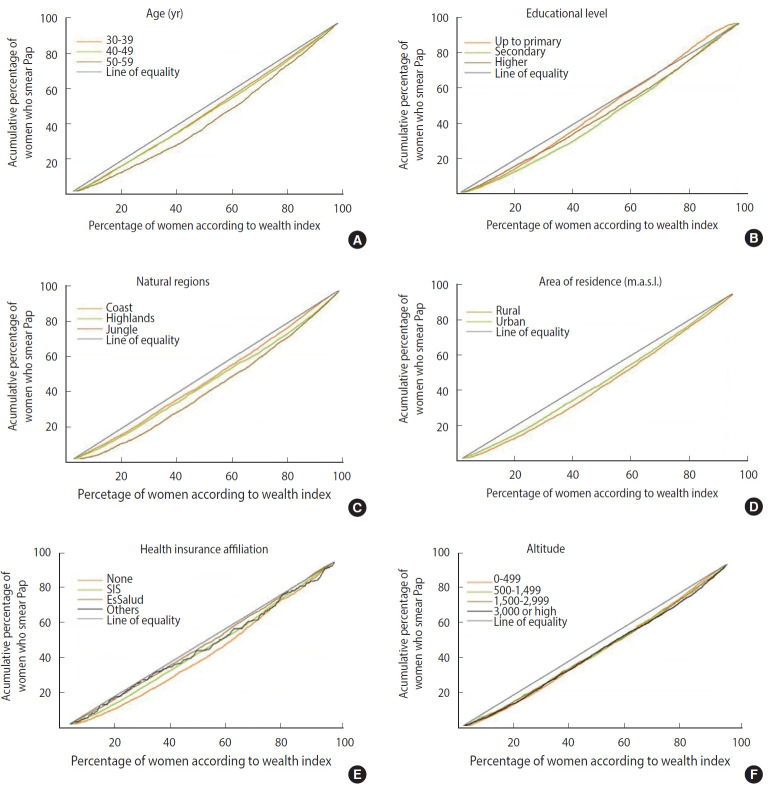
Concentration curves of Pap smear uptake in relation to the characteristics (A,: age, B: educational level, C: natrual regions, D: area of residence, E: health insurance affiliation, and F: altitude) of the population during the 2015-2017. SIS, Seguro Integral de Salud; m.a.s.l., meters above sea level.

CCs and IndCs were constructed for the same variables. Most of the CCs were below the equality line, meaning that uptake of the Pap test was concentrated in the population of women with higher wealth indices (Figure 2). All the values obtained from the IndC were greater than 0, reaffirming that the performance of the Pap test was concentrated in the population with higher wealth indices (Table 3). The highest IndC values (highest inequality) were found among women who were 50-59 years of age (IndC, 0.293), those residing in the jungle (IndC, 0.230), and women not affiliated with any health insurance (IndC, 0.173); while the lowest IndC values were found in women with up to primary education (IndC, 0.043), those affiliated with EsSalud (IndC, 0.044), and women residing on the coast (IndC, 0.090).

## DISCUSSION

In this study, we sought to analyze the socio-demographic inequalities in the uptake of the Pap test among Peruvian women. We found that only 60.4% of those surveyed between the years 2015-2017 had undergone a Pap test in the 3 years prior to the survey. The most relevant characteristics for a higher uptake of the Pap test were: having health insurance affiliated with EsSalud, belonging to the highest wealth quintile, having higher education, and living on the coast. In contrast, lower Pap test uptake was observed among women belonging to the lowest wealth quintile, without any health insurance, women from 50 years to 59 years of age, and those living in the jungle. The CCs and IndCs showed that Pap smear uptake was consistently concentrated amongst the population with a higher wealth index, thereby demonstrating inequality.

Although the proportion of women who had undergone a Pap test in the 3 years prior to the survey had increased from 53.9% in 2013 [24], according to our findings, the rate remained low at only 60.4%. This number is below the coverage achieved in European countries such as Spain (70.2%) [25] and Italy (62.1%) [26], and in other South American countries including Colombia (82.9%) [27] and Brazil (79.4%) [28]. The World Health Organization (WHO) has proposed that in order to eliminate cervical cancer as a public health problem, a minimum coverage of 70% with high-precision tests should be achieved [29]. Sadly, in Peru, the only test studied nationwide is the Pap test, with which we are still far from reaching this goal. Further work is needed to close the gaps that affect Pap test uptake and to improve national coverage.

We found that women aged 50-59 had a higher level of inequality with regard to Pap test uptake than women aged 30-39 (IndC, 0.293 vs. 0.171, respectively). The same tendency has been observed in Spain, with Pap smear uptake decreasing from 72.5% among women aged 25-35 to 61.5% in those from 53 years to 65 years of age [25]. The higher inequalities observed in older women might be explained by a reduction in routine medical check-ups. Previous studies suggest that younger women are more likely to be exposed to healthcare centers for prenatal or pediatric care for their young children [11,30,31]. Furthermore, it is likely that older women are not aware of the impact that a Pap test has for combating cervical cancer, and instead prioritize other chronic health problems that increase in incidence with age, leaving aside screening practices.

The natural region of residence was another important characteristic related to uptake of the Pap smear. There was a notable difference among women living in the jungle and those residing on the coast (IndC, 0.230 vs. 0.090, respectively). This is likely due to the greater concentration of specialized infrastructure and human health resources and the greater accessibility to healthcare centers in coastal cities such as Lima, Arequipa, and Callao [32,33] in comparison to other regions where the uneven landscape and natural barriers make access to health services difficult. In the jungle, it is common for the rural population to travel by river to access healthcare; this can take up to several days and may be more difficult in certain months of the year due to changing water levels, which in some cases do not allow or significantly hinder navigation [34-36]. In a similar vein, a study conducted in women attending the outpatient clinic of a hospital in Uganda found that up to 32.9% of women had never been screened because they did not live near a healthcare center [37]. Likewise, Collins et al. [34] found that, despite having insurance, women who lived in isolated communities in the Peruvian jungle must assume additional expenses, such as transportation, to access health services, thereby limiting their access. The presence of geographic barriers increases the inequality in this at-risk population; therefore, strategies such as a higher number of screening campaigns within communities and mobile clinics, which have already been implemented in some parts of the territory [38], should be extended to reduce these gaps.

Women who were not affiliated with health insurance had a higher IndC (greater inequality) than those affiliated with EsSalud (IndC, 0.173 vs. 0.044, respectively). This is consistent with studies conducted in China and Colombia where Pap smear uptake among uninsured women was 14.4% and 10.3%, respectively [11,39]. Even though health care system reforms have reduced inequalities in access to health insurance, in 2017 it was reported that nearly a quarter of the population (24.5%) still did not have health insurance [40]. Not having health insurance causes this population to have greater out-of-pocket expenses related to health. The WHO estimates that 100 million people fall into extreme poverty each year due to health expenses [41]; hence, uninsured women are especially vulnerable. This is not the case for women affiliated with EsSalud, who have a stable job and more resources to face health expenses. Barrionuevo-Rosas et al. [19] reported that Peruvian women with public or private insurance were more likely to receive Pap smears (prevalence ratios, 1.27 and 1.52, respectively) than those without insurance. Nonetheless, the consolidation of universal health insurance that will allow all Peruvians equal access to health services is still pending.

We found that the least inequality in the uptake of the Pap test was among women with only up to primary education, with far greater inequality found among women with up to secondary education (IndC, 0.043 vs. 0.161, respectively). This should not be interpreted as a reduction in the gap of Pap smear uptake. On the contrary, these women had the lowest Pap smear uptake overall, with fewer than half of the women in this group (49.5%) having undergone a Pap smear in the previous 3-year. It is to be expected that women with a lower education level will have less knowledge about cervical cancer and its prevention, as well as fewer resources to face health problems. In their systematic review, Islam et al. [42] found that despite small variations in barriers for cervical cancer screening according to the socio-demographic characteristics of the population, it was lack of knowledge about cervical cancer and screening practices that acted as the main barrier for uptake. Women who have only a basic understanding of what goes on in the world are unable to exercise their autonomy and often depend on misinformed comments and myths about important topics such as cancer screening, thereby limiting screening uptake and hindering decision-making that would allow them to improve their health.

Finally, although the differences in the IndC were not as marked as those in the other variables, we found that there was greater inequality in the uptake of the Pap test in women who lived in rural areas and within 500 m. a. s. l. On one hand, there is a higher density of doctors in urban coastal areas such as Lima and Callao, with a rate of 20.5 and 22.5 doctors per 10,000 inhabitants, respectively. These numbers are almost triple those in other departments located at the same altitude in other natural regions, such as Loreto and Ucayali, which are located in the jungle and have 7.2 and 8.3 doctors per 10,000 inhabitants, respectively [33]. In addition, there is a centralized distribution of health facilities and less availability of infrastructure aimed at the care of oncological diseases. These are mainly concentrated in Lima, which has up to 30 times more health facilities than other departments [38,43]. In Peru, living in rural areas and in geographically isolated regions limits both the availability of human health resources and access to adequate sanitary infrastructure.

The main limitations of this study are the inability to establish causal associations or include other variables of interest because of the cross-sectional, secondary analytical nature of the study. It should also be mentioned that since the data collected in the survey are self-reported, there may be the risk of recall and social desirability bias. In addition, possible omissions or registry errors in the survey could influence its reliability. Nevertheless, ENDES is designed to systematically compile information about demographic and health characteristics at a population-based level and has trained staff to achieve this goal. Currently, ENDES is the only available source of information about Pap smear uptake in the Peruvian territory. The inequality that our population faces cannot be described in its entirety by a single study. However, the main socioeconomic variables that were associated with the uptake of Pap tests were studied together with some geographic variables in the Peruvian context.

In conclusion, we found socio-demographic inequalities in the uptake of Pap smear in Peru, with the population with a higher wealth index receiving the most benefits. These disparities have persisted despite reforms in the healthcare system and the implementation of programs to decrease the gaps in access to cervical cancer screening. Further investment in health programs and the implementation of systems that ensure uniform access to health in our country are needed in order to reduce the loss of life-related to cervical cancer in the Peruvian population.

## Figures and Tables

**Table 1.** Characteristics of the women included in the 2015-2017 Demographic and Family Health Survey (ENDES, acronym in Spanish) surveys

Characteristics	Frequency (n=27,991)	% (95% CI)^1^
Age (yr)		
30-39	14,774	40.6 (39.7, 41.5)
40-49	7,875	33.3 (32.4, 34.2)
50-59	5,342	26.1 (25.2, 26.9)
Mean±SD	40.7±8.3	
Highest educational level reached		
Up to primary	10,221	35.8 (34.5, 37.0)
Secondary	9,845	35.0 (33.9, 36.1)
Higher	7,925	29.2 (28.0, 30.4)
Wealth quintile		
I (lowest)	7,117	21.6 (20.4, 23.9)
II	6,980	20.5 (19.5, 21.5)
III	5,626	19.6 (18.7, 20.5)
IV	4,747	20.0 (19.0, 21.1)
V (highest)	3,521	18.3 (17.0, 19.6)
Natural region of residence		
Coast	11,774	55.3 (53.1, 57.5)
Highlands	9,631	30.7 (28.7, 32.7)
Jungle	6,586	14.0 (12.8, 15.4)
Area of residence		
Rural	8,701	28.3 (27.0, 29.8)
Urban	19,290	71.7 (70.2, 73.0)
Altitude relative to sea level of the housing conglomerate (m. a. s. l.)		
0-499	13,537	56.4 (54.3, 58.5)
500-1,499	3,364	8.1 (7.1, 9.3)
1,500-2,999	4,130	13.1 (11.7, 14.6)
≥3,000	6,960	22.3 (20.8, 24.0)
Type of health insurance		
Seguro Integral de Salud	14,762	47.7 (46.4, 48.9)
EsSalud	7,276	28.2 (27.2, 29.3)
Others	439	2.6 (2.2, 3.0)
None	5,514	21.5 (20.6, 22.4)

CI, confidence interval; SD, standard deviation; m.a.s.l., meters above sea level.

1 Weighted proportion.

**Table 2.** Socio-demographic characteristics in relation to the uptake of Pap smears among the women surveyed during the 2015-2017 Demographic and Family Health Survey (ENDES, acronym in Spanish) surveys

Characteristics	Papanicolaou smear, % (95% CI)^1^		p-value^2^
	No	Yes	
Age (yr)			
30-39	33.7 (32.4, 34.9)	66.3 (65.1, 67.6)	<0.001
40-49	37.9 (36.3, 39.6)	62.1 (60.4, 63.7)	
50-59	51.0 (48.9, 53.1)	49.0 (46.9, 51.1)	
Highest educational level reached			
Up to primary	50.5 (48.9, 50.0)	49.5 (48.0, 51.1)	<0.001
Secondary	36.5 (34.9, 38.0)	63.5 (62.0, 65.1)	
Higher	30.1 (28.5, 31.7)	69.9 (68.3, 71.5)	
Wealth quintile			
I (lowest)	51.6 (49.6, 53.6)	48.4 (46.4, 50.4)	<0.001
II	44.1 (42.3, 45.9)	55.9 (54.1, 57.7)	
III	37.1 (35.2, 39.1)	62.9 (60.9, 64.8)	
IV	34.6 (32.8, 36.6)	65.4 (63.4, 67.2)	
V (highest)	28.5 (26.2, 31.0)	71.5 (69.0, 73.8)	
Natural region of residence			
Coast	34.5 (33.1, 35.8)	65.5 (64.2, 66.9)	<0.001
Highlands	44.7 (43.0, 46.3)	55.3 (53.7, 57.0)	
Jungle	48.9 (46.7, 51.0)	51.1 (49.0, 53.3)	
Area of residence			
Rural	47.9 (46.0, 49.8)	52.1 (50.2, 54.0)	<0.001
Urban	36.3 (35.2, 37.5)	63.7 (62.5, 64.8)	
Altitude relative to sea level of the housing conglomerate (m. a. s. l.)			
0-499	36.3 (34.9, 37.6)	63.7 (62.4, 65.1)	<0.001
500-1,499	40.0 (37.3, 42.7)	60.0 (57.3, 62.7)	
1,500-2,999	40.0 (37.8, 42.2)	60.0 (57.8, 62.2)	
≥3,000	47.7 (45.8, 49.6)	52.3 (50.4, 54.2)	
Type of health insurance			
Seguro Integral de Salud	41.7 (40.3, 43.0)	58.3 (57.0, 59.7)	<0.001
EsSalud	25.6 (24.1, 27.2)	74.4 (72.8, 75.9)	
Others	32.4 (26.1, 39.5)	67.6 (60.5, 73.9)	
None	54.3 (52.3, 56.3)	45.7 (43.7, 47.7)	

m.a.s.l., meters above sea level.

1 Weighted proportion.

2 Chi-square test.

**Table 3.** Concentration index of Pap smear uptake according to the characteristics of the women surveyed during the 2015-2017 Demographic and Family Health Survey (ENDES, acronym in Spanish) surveys

Characteristics	Concentration index	p-value^1^
Age (yr)		
30-39	0.171	<0.001
40-49	0.182	
50-59	0.293	
Highest educational level reached		
Until Primary	0.043	<0.001
Secondary	0.161	
Higher	0.136	
Natural region of residence		
Coast	0.090	<0.001
Highlands	0.133	
Jungle	0.230	
Area of residence		
Rural	0.166	0.012
Urban	0.124	
Altitude about sea level of the housing conglomerate (m. a. s. l.)		
0-499	0.133	<0.001
500-1,499	0.118	
1,500-2,999	0.122	
≥3,000	0.127	
Type of health insurance		
Seguro Integral de Salud	0.104	<0.001
EsSalud	0.044	
Others	0.085	
None	0.173	

m.a.s.l., meters above sea level.

1 Fisher test.
